# The *Cool Little Kids *randomised controlled trial: Population-level early prevention for anxiety disorders

**DOI:** 10.1186/1471-2458-11-11

**Published:** 2011-01-05

**Authors:** Jordana K Bayer, Ronald M Rapee, Harriet Hiscock, Obioha C Ukoumunne, Cathrine Mihalopoulos, Susan Clifford, Melissa Wake

**Affiliations:** 1 Department of Pediatrics, The University of Melbourne, Parkville, Australia; 2 Murdoch Childrens Research Institute, Parkville, Australia; 3 Centre for Community Child Health, Royal Children's Hospital, Parkville, Australia; 4 Center for Emotional Health, Macquarie University, North Ryde, Australia; 5 Clinical Epidemiology and Biostatistics Unit, Royal Children's Hospital, Parkville, Australia; 6 Deakin University, Burwood, Australia

## Abstract

**Background:**

The World Health Organization predicts that by 2030 internalising problems (e.g. depression and anxiety) will be second only to HIV/AIDS in international burden of disease. Internalising problems affect 1 in 7 school aged children, impacting on peer relations, school engagement, and later mental health, relationships and employment. The development of early childhood prevention for internalising problems is in its infancy. The current study follows two successful 'efficacy' trials of a parenting group intervention to reduce internalising disorders in temperamentally inhibited preschool children. *Cool Little Kids *is a population-level randomised trial to determine the impacts of systematically screening preschoolers for inhibition then offering a parenting group intervention, on child internalising problems and economic costs at school entry.

**Methods/Design:**

This randomised trial will be conducted within the preschool service system, attended by more than 95% of Australian children in the year before starting school. In early 2011, preschool services in four local government areas in Melbourne, Australia, will distribute the screening tool. The ≈16% (n≈500) with temperamental inhibition will enter the trial. Intervention parents will be offered *Cool Little Kids*, a 6-session group program in the local community, focusing on ways to develop their child's bravery skills by reducing overprotective parenting interactions. Outcomes one and two years post-baseline will comprise child internalising diagnoses and symptoms, parenting interactions, and parent wellbeing. An economic evaluation (cost-consequences framework) will compare incremental differences in costs of the intervention versus control children to incremental differences in outcomes, from a societal perspective. Analyses will use the intention-to-treat principle, using logistic and linear regression models (binary and continuous outcomes respectively) to compare outcomes between the trial arms.

**Discussion:**

This trial addresses gaps for internalising problems identified in the 2004 World Health Organization Prevention of Mental Disorders report. If effective and cost-effective, the intervention could readily be applied at a population level. Governments consider mental health to be a priority, enhancing the likelihood that an effective early prevention program would be adopted in Australia and internationally.

**Trial Registration:**

ISRCTN: ISRCTN30996662

**RCH Human Research Ethics Approval:**

30105A

## Background

Few people in modern societies are untouched by internalising problems, a broad term that refers to emotional distress and encompasses the spectrum of emotional symptoms of anxiety and depression. Although in clinical practice anxiety and depression disorders are seen as multiple, distinct diagnoses, empirical evidence shows high overlap between them and supports use of the broad term internalising problems [1,2]. The World Health Organization (WHO) predicts that, by 2030, internalising problems will be second only to HIV/AIDS in burden of disease [3].

Mental health problems affect 1 in 7 school aged children [4], although they can occur in children of all ages. Internalising (emotional) and externalising (behavioural) problems are among the most common difficulties of early childhood, affecting approximately 15% of those aged 18 months to 5 years [5-8]. Australian community studies have recently confirmed this high prevalence and stability of internalising symptoms across early to mid childhood (e.g. Pearson r's = .53 to .63) [9,10]. By the time internalising disorders are detected problems can be severe and treatment effectiveness can be limited [11,12].

Early internalising problems often have longer-term consequences, with many adult problems having early roots in childhood [4,13,14]. Convergent evidence from prospective and retrospective studies confirms that internalising problems often persist into adolescence and then into adulthood [4,15-24]. Their impacts extend beyond mental health to adult relationships, employment opportunities, and even early mortality. For example, in the British National Child Development Study (N = 11,142), internalising problems at ages 7-11 years were predictive of higher mortality by age 45 (OR 1.20, 95% CI 1.06-1.35) [21].

Evidence suggests that early intervention is key to producing a positive impact because it may be more difficult to influence developmental outcomes later in childhood [25,26]. Though limited, the evidence also supports the cost-effectiveness of intervening early in development [25,27,28]. While emotional functioning continues to develop into adulthood, the early years constitute a window of opportunity for effective mental health promotion in at-risk children. The application of prevention to internalising problems in early childhood is still in its infancy [29]. In 2009, Bayer and colleagues conducted a systematic review of early interventions (age 0-8 years) to improve child mental health. This review found a paucity of randomised controlled trials aiming to reduce internalising problems in community settings [30].

### Rationale for the proposed *Cool Little Kids *population-level study

The strongest precursor of internalising problems in young children is temperamental inhibition, manifested as fearfulness and a tendency to withdraw from new situations [29,31-34]. Additional known risks are harsh and overprotective parenting, and parent internalising problems [9,10,34-39]. Together, these account for up to 45% of the variance in early childhood internalising symptoms [9,10,35].

The only randomised trials testing a parenting prevention model in inhibited preschool children were conducted by Rapee. Rapee's *Cool Little Kids *program is the first (and, thus far, only) effective early childhood prevention program for internalising disorders [30,40-42]. Targeting child inhibition and overprotective parenting, this parenting program aims to help preschool children become resilient to situational fears and abstract distressing worries. It teaches parents strategies to modify their preschool child's fear and distress, as well as their own (if relevant), based on standard principles for treating internalising disorders in children and adults [11,42].

Two successful efficacy trials of the *Cool Little Kids *program have been reported. Rapee's first trial [41] recruited 146 children aged 4 years with temperamental inhibition, measured by parent-report questionnaire (>85th percentile) and intensive laboratory observation. Intervention parents received a university-based prevention program from teams of two clinical psychologists offering six group sessions designed to reduce overprotective parenting in response to early fearful behaviour. By age 5 years, the intervention children had developed significantly fewer anxiety disorders than controls (50% vs. 64%). These effects were even larger by age 7 (40% vs. 69%) [42]. Rapee's second study [40] recruited 71 inhibited preschoolers whose parents themselves had internalising disorders. The intervention group received an eight-session version of the program which extended to focus on parent anxiety as well as overprotective parenting. Six months later, the intervention children had substantially fewer internalising disorders, diagnosed in only 53% of the intervention group compared to 93% of controls.

Rapee's two efficacy trials are at the cutting edge of prevention research and have major potential public health implications. Population conclusions, however, are precluded by their sample bias (university location and self-selection by advertisement) and the labour-intensive laboratory observation methods used for selection. The unaddressed challenge is to determine 'real world' effectiveness across an entire population. We report the protocol for the next step - to conduct a population-level translational randomised trial.

### Aims and hypotheses

The aims of this trial are to (a) determine the balance of benefits and harms of systematically screening preschoolers for temperamental inhibition and of a parenting intervention program offered to those at risk, (b) examine the impacts on child internalising problems at school entry, and (c) evaluate cost-effectiveness.

We hypothesise that children whose parents enter the program will do better one and two years after baseline (the first two years of school for most) than 'usual care' control children on the outcomes: i) fewer children with internalising disorders, ii) lower mean scores on early child internalising symptoms, iii) lower mean scores on harsh and overprotective parenting, iv) lower mean scores on parent internalising problems. We anticipate that the prevention program will be acceptable and cost-effective.

## Methods/Design

### Overview of Methods

**Figure 1:**
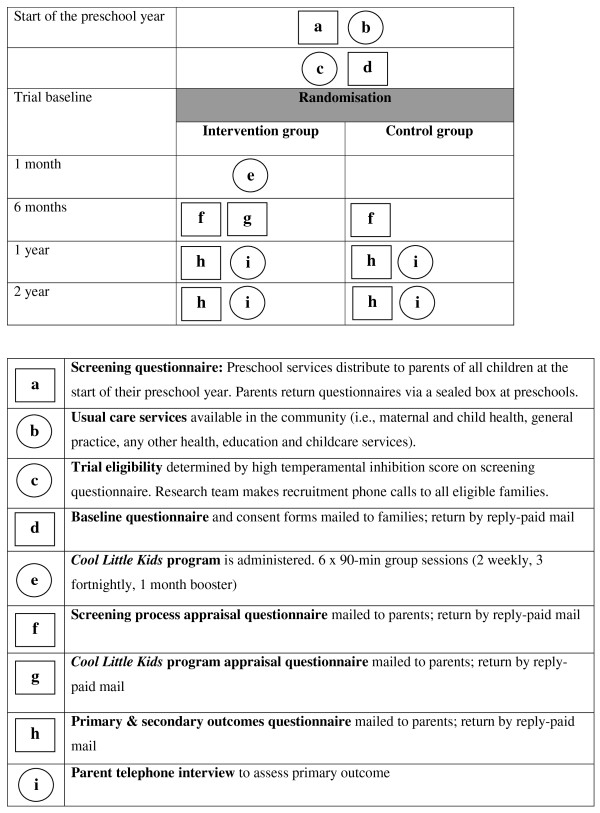
**Graphical depiction of components of the trial**.

Figure 1 summarises the time line of the trial. It shows graphically the features at each stage that are common to both the intervention and control groups, and unique to the intervention group, in the manner suggested by Perera *et al*. [43].

*Cool Little Kids *is prospectively registered with an international clinical trials registry (ISRCTN30996662), will be conducted in line with ISPOR guidelines for cost-effectiveness trials alongside RCTs [44], and reported in accordance with the CONSORT statement. Project approval has been obtained from the Ethics in Human Research Committee of the Royal Children's Hospital, Melbourne, Australia

### Recruitment and participants

Extending *Cool Little Kids *to a population randomised trial requires more than evidence supporting intervention effectiveness. It requires 1) a universal service system attended by all or almost all 4 year olds, and 2) an acceptable screening tool to systematically identify the children at risk. Primary health care settings were not considered to be an appropriate setting for this screening, because in many countries health practitioners lack the time and resources to screen for this type of problem. In Australia, preschool services are accessed by almost all 4 year olds (95%) [45], at a time when parents and early childhood teachers are concerned about children's impending readiness for school [46]. Brief universal screening can therefore be reliably placed in the preschool setting. The known risk factors for internalising problems are child temperamental inhibition, parents' own internalising problems and overprotective and harsh discipline parenting. The potential benefit versus harm of screening will therefore be considered. For preschool children, it is likely to be more acceptable both to parents and the educational preschool setting to screen inhibition as a child precursor of internalising problems, rather than their parents' mental health and parenting characteristics.

Preschool services from four government areas will be selected to span the sociodemographic spectrum. Written and verbal briefing (study aims, recruiting procedure) will be delivered to preschools across local government areas in the second half of 2010. Recruitment will take place in early 2011 over the first few months of the preschool year. Preschools will distribute a study package including an Information Statement with a screening and consent questionnaire to all parents of children enrolled in their year prior to school. Parents will return this screening and consent questionnaire in a confidential envelope to a sealed letter box at their child's preschool. The questionnaire includes The Short Temperament Scales [47] to screen for child temperamental inhibition (see Table 1) and demographic items.

**Table 1:** Measures used in analysis of study outcomes

Construct	Measure	Administration time points	Number of items	Additional Information
***Child measures***				

Temperamental inhibition	Short Temperament Scales - Inhibition subscale [47]	Screening	7	Children who score ≥85^th ^percentile are eligible for the trial.

Major health or developmental diagnoses	Parents' Evaluation of Developmental Status (Australian version)[53]	RCT baseline	10	Children with major diagnoses will be excluded: determined on a case-by-case basis.

Behavioural and emotional problems	Strengths & Difficulties Questionnaire (4-10 year old Australian version)[54]	Screening 1 & 2 years	25	Behavioural screening measure; widely used in population health research; existing school-entry screen in Victoria. Emotional subscale may have concurrent validity with temperamental inhibition screen. Secondary outcome (impact on externalising/conduct problems).

Anxiety diagnoses	Anxiety Disorders Interview Schedule for DSM-IV, Child Version, Parent Interview Schedule (ADIS-CP-IV) [55]	1 & 2 years		Primary outcome. Conducted by telephone interview.

Internalising problems	Children's Moods, Fears and Worries Questionnaire [1]	1 & 2 years	34-38	Primary outcome. Detailed measure of internalising symptoms (anxious, fearful, withdrawn, depressive) in young children.

Anxiety problems	Preschool Anxiety Scale - Revised (PAS-R)[56]	1 & 2 years	28	Primary outcome. Detailed measure of anxiety symptoms in young children. Sensitive to intervention in *Cool Little Kids *efficacy trial.

***Parent measures***				

Parenting practices	Parent Behavior Checklist (nurturing, harsh discipline) [57] Over-involved/protective parenting [10,35]	Baseline 1 & 2 years	32 8	Secondary outcome (intervention mechanism for impacting on child).

Mental health	Depression, Anxiety, Stress Scale [58]	Baseline 1 & 2 years	21	Secondary outcome (impact on parent wellbeing).

Wellbeing	SF-12 [59]	Baseline	12	Secondary outcome (economics measure).
	Assessment of Quality of Life (AQoL-8D) [49]	1 & 2 years	35	Secondary outcome (economics measure).

***Intervention 'process' measures***				

Fidelity	Group sessions content checklists, rated by facilitator and researcher	6 month appraisal	6-8	Secondary outcome (integrity of intervention delivery). Adapted for this study.

Acceptability to parents	Perceptions of screening process Perceptions of group sessions content, group facilitator	6 month appraisal	4 15	Secondary outcome (implications for translation/dissemination uptake by families). Adapted for this study.

Cost-effectiveness	Child and adult health service use	1 & 2 years	6	Secondary outcome (for policy/decision makers considering translation/dissemination). Generated for this study.

After scoring, the study team will notify parents by letter if their child does not score highly on temperamental inhibition and is therefore ineligible for the trial. Eligible parents will be mailed the detailed trial Information Statement and Consent form with the baseline questionnaire ascertaining family risk for internalising problems and more detailed demographic characteristics (see Table 1). The study team will also telephone all eligible parents to explain trial procedures in detail and answer parents' questions.

### Selection criteria

#### Inclusion criteria

Parents will be eligible for the universal screening component of the study (completing the brief parent-report questionnaire) if their child is enrolled for their preschool year (i.e. the year prior to school) at a participating preschool/kindergarten service. They will be eligible to join the trial if the child scores ≥85^th ^percentile on the inhibition subscale of the Short Temperament Scales [47].

#### Exclusion criteria

Exclusion criteria are (1) parents with insufficient English to participate (determined by parent or teacher report, or by the study team following up on insufficiently completed screening questionnaires) and (2) children with major health or developmental problems who are considered unlikely to benefit from the intervention. Major health or developmental problems will be discussed on a case-by-case basis, which mirrors what would happen when subsequently translated to the broader population. As exclusion occurs before randomisation, it in no way affects the trial's internal validity.

### Allocation

After recruitment and consent, the statistician will coordinate a concealed web-based randomisation process. Computer-generated sequences of random numbers will be used to determine the trial arm status of each child. A block randomisation process, stratified for each preschool separately, will minimise the imbalance between the numbers of intervention and control participants within each preschool. After families are randomised, the study team will notify all parents by letter whether they are in the intervention or control arm, and arrange group session bookings for intervention parents. Participant allocation will be concealed to the clinician assessing diagnostic outcomes; however, the group facilitators and parents cannot be blinded to their group allocation.

### Intervention

#### Content

For this population-level randomised trial, the *Cool Little Kids *parenting intervention consists of manualised parenting group sessions as previously tested in Rapee's efficacy research [41]. Parents will receive a 'workbook' at the first parenting session that presents information on the nature of inhibition, fears, anxiety and emotional distress, and the developmental trajectory of internalising problems. Thereafter, it details instructions in exposing children to their specific triggers for emotional distress to develop reality-testing and coping skills. In addition, it presents effective ways of parenting inhibited children, and methods for parents to manage their own worries and distress. The latter component involves parents applying strategies for children to themselves, plus a section on cognitive restructuring. Parents are taught how to think more realistically to manage their own concerns, so that they can gradually introduce cognitive techniques to their children as they grow older. The manual is supplemented with extensive examples and detailed exercises. It also contains information on adult mental health problems and encourages parents to seek professional help if relevant. Families with low literacy, and culturally and linguistically diverse families, will be supported by using plain English content.

#### Process - Intervention group

The intervention includes six group sessions of 90 minutes. These sessions focus on the principles outlined in the manual. Sessions involve setting readings from the manual, scheduling and motivating implementation and practice, and trouble-shooting difficulties arising during practice at home. The first two group sessions are one week apart to ensure comprehension of materials, discuss motivation and encourage implementation of the earliest strategies. The next three sessions are at fortnightly intervals to allow parents time to implement strategies and to encounter any difficulties. The final session is a booster session four weeks later to motivate parents for their longer-term goals and incorporate strategies into broader aspects of family life.

Groups of parents (~12 individuals) with inhibited children will be offered the *Cool Little Kids *program, delivered by early childhood professionals skilled in cognitive behavioural therapy techniques and instructed in the program by accredited trainers. Parent groups will be run at one of the participating preschools, or another local venue (such as a local maternal and child health service) that is convenient and acceptable to parents.

#### Process - Control group

Families in the control arm will receive usual teaching and care from their preschool, early childhood and health services in the community. This may include advice on children's behaviour, but would not include a structured, evidence-based parenting program for temperamental inhibition or internalising problems.

### Outcome measurement

Approximately one (2012) and two (2013) years after baseline, a clinician blind to group allocation will interview parents to ascertain child internalising diagnoses (see Table 1). These interviews will be conducted by telephone, which has demonstrated validity and is less expensive than face-to-face interviews [48]. A questionnaire mailed to parents will further measure child internalising symptoms, parenting practices, parent mental health and service use.

### Sample size

The randomised trial will have a sample size of around 500 inhibited children, providing 80% power at the 5% level of significance to detect the level of reduction in internalising problems found in Rapee's prior efficacy trials, namely a 14% reduction in child internalising disorders between the intervention (50%) and control (64%) groups [41]. This sample size estimation considers an achievable attrition rate for a quality trial, allowing for up to 20% loss to follow up.

### Data analyses

Regression analyses will compare child outcomes and costs at school-entry (1 and 2 years after baseline) between the intervention and control arms using the intention-to-treat principle, where participants are analysed in the groups to which they were randomised. Logistic regression will be used for binary outcomes (presenting the percentages for each trial arm and odds ratio between them) and linear regression for continuous outcomes (presenting the mean and standard deviation for each trial arm and mean difference between them). Both unadjusted analyses and analyses adjusted for potential confounding factors determined *a priori *before randomisation (child gender, family socioeconomic level, parent mental health) will be implemented. Tests of interaction, specified *a priori*, will explore differential intervention effects for children who are higher on risk factors at baseline. In line with Rapee's second efficacy trial [40], we anticipate larger group differences might emerge for children who are higher on risk factors at baseline.

### Process evaluation

Parents and group facilitators will also be mailed questionnaires at the end of the intervention (late 2011) to evaluate the screening process and intervention, where applicable. All parents will report on perceived harms and benefits of screening. Intervention parents and group facilitators will report on intervention acceptability, extent to which components were implemented, and barriers to attendance. Group facilitators will complete a standard content checklist after each group session to record the degree to which the intervention was delivered. In addition, a research assistant will observe a random 10% of parent groups and evaluate protocol adherence using the content checklist.

### Economic Evaluation

Economic evaluation will also be conducted to determine whether population application of *Cool Little Kids *provides value for money to governments (if dissemination were publicly funded), families and society in general. Economic evaluation is a comparative technique of an intervention's costs and consequences to a comparator's costs and consequences (in this instance the control group) measured as an incremental cost-effectiveness ratio (ICER). Since the current study employs a range of outcomes, the primary study design is that of a cost-consequence analysis (CCA). Such an analysis captures all relevant study outcomes which are subsequently presented to decision-makers, allowing them to make their own trade-offs regarding which are more important than others.

Most of the study outcomes are largely clinical in nature (e.g. diagnosis of anxiety disorders, scores of the various clinical scales). This means that the majority of analyses will be cost-effectiveness analyses (CEA). While such analyses are important they have limitations in deciding whether the costs and benefits of interventions represent good value-for-money or not. Cost-utility analysis (CUA) is a form of cost-effectiveness analysis where the costs are measured in monetary terms and the consequences in a generic outcome metric capable of capturing both mortality and morbidity effects, allowing judgments regarding the 'worth' or the 'value' of interventions to be made (as well as comparisons to other interventions both within and across the different disorder/diseases). Parental utility will be measured using the Assessment of Quality of Life Scale -8 Dimension (AQoL8D) [49]. There are no existing tools to measure utility in preschool children, though as the children age measures such as the Health Utilities Index 3 (HUI) may be used [50]. Alternatively, "proxy" utilities may need to suffice as used in a recent study which modeled the efficacy credentials of the *Cool Little Kids *intervention [51]. It is because the economic analyses will be comprised of both CEA and CUA analyses, the appropriate study-frame is a cost-consequence analysis.

The underlying principle in identifying costs relevant to the economic evaluation is that the inclusion/exclusion criteria should mirror the study perspective. This economic evaluation intends to adopt a largely societal perspective, so all costs to the health sector, participants and their families as well as other sectors impacted upon by the intervention will be included. As there are no previous economic evaluations in this area, a comprehensive resource use questionnaire is proposed to capture a wide variety of resource use for both parents and children (including time costs of parents). Costs associated with the initial research, design and set-up of the intervention and the development of any program materials will be excluded, as these are largely one-off 'sunk' costs (unrecoverable past expenditures), and will not be incurred in on-going routine implementation of the intervention.

The technique of 'bootstrapping' will be used to obtain confidence intervals for cost effectiveness ratios, since parametric techniques are inappropriate for use on both skewed variables and ratios. The sensitivity of the results will also be tested against: different discounting scenario; variation in the utility weights for the children; and, differences in unit cost prices. Costs falling upon the health sector, patients or their families, the government and other sectors will be presented in total and disaggregated form.

The value of economic evaluations alongside effectiveness trials is that a comprehensive analysis of the costs and benefits at an individual level can be obtained. However, such evaluations are usually time limited and do not capture the longer term costs and benefits associated with such interventions. Modelling techniques are a valuable addition to trial based evaluations. Firstly, the longer term costs and consequences associated with interventions can be estimated, though such analyses unavoidably require some assumptions to be made (e.g. a sustained effect of the intervention beyond the duration of the trial). Secondly, modelling allows the costs and benefits at a population level to be estimated. Such information is important to decision makers who are set with the task of allocating national health care budgets and must choose between different interventions. Such modelling of the current intervention is feasible and would build upon a previous study which evaluated the population cost-effectiveness of the *Cool Little Kids *intervention [51].

## Discussion

This translational randomised trial aims to determine 'effectiveness' of the *Cool Little Kids *program administered across the population. Rapee's recent efficacy studies will be extended to:

a) determine the balance of benefits and harms of systematically screening preschoolers for temperamental inhibition

b) determine the balance of benefits and harms of a parenting intervention program offered from a universal preschool platform to all at risk children in the year prior to school,

c) examine the impacts on internalising problems at school entry, a key developmental point of considerable policy interest internationally,

d) include time-efficient and valid screening and outcome assessments including parenting practices and parent mental health (in addition to child internalising problems), and

e) evaluate cost-effectiveness, to inform policy and service delivery.

An outcome of this translational trial could be systematic screening leading to routine prevention for all or most preschoolers at risk, building on existing universal preschool and healthcare systems. If the *Cool Little Kids *program proves effective at population level, large-scale rollout is appropriate. A population approach to preventing internalising problems early in childhood will address gaps identified in the WHO Prevention of Mental Disorders Report [52]. It would be highly innovative to prevent internalising problems early in life, in contrast to current timing of mental health service entry, typically in adolescence after anxiety and depression become entrenched. Reducing child internalising problems early in life should subsequently narrow cumulative disparities in mental health and related disadvantage later in life. In Australia and internationally, governments currently consider mental health and early prevention to be a priority.

## Competing interests

The authors declare that they have no competing interests.

## Authors' contributions

JB took the leading role in designing the study and writing the grant that was subsequently funded by the Australian National Health and Medical Research Council (NHMRC) and modifying the grant for publication. RR developed the *Cool Little Kids *parenting intervention. RR, MW, HH, OU and CM contributed to the study design and grant preparation. SC assisted with the manuscript. All authors read and approved the final manuscript.

## Pre-publication history

The pre-publication history for this paper can be accessed here:

http://www.biomedcentral.com/1471-2458/11/11/prepub
